# Microvasculature-directed thrombopoiesis in a 3D *in vitro* marrow microenvironment

**DOI:** 10.1371/journal.pone.0195082

**Published:** 2018-04-04

**Authors:** Surya Kotha, Sijie Sun, Amie Adams, Brian Hayes, Kiet T. Phong, Ryan Nagao, Jo-Anna Reems, Dayong Gao, Beverly Torok-Storb, José A. López, Ying Zheng

**Affiliations:** 1 Department of Bioengineering, University of Washington, Seattle, Washington, United States of America; 2 Bloodworks Research Institute, Seattle, Washington, United States of America; 3 Clinical Research Division, Fred Hutchinson Cancer Research Center, Seattle, Washington, United States of America; 4 Department of Mechanical Engineering, University of Washington, Seattle, Washington, United States of America; 5 Department of Medicine (Hematology), University of Washington, Seattle, Washington, United States of America; 6 Center for Cardiovascular Biology, and Institute of Stem Cell and Regenerative Medicine, University of Washington, Seattle, Washington, United States of America; University of Minnesota Medical Center, UNITED STATES

## Abstract

Vasculature is an interface between the circulation and the hematopoietic tissue providing the means for hundreds of billions of blood cells to enter the circulation every day in a regulated fashion. The precise mechanisms that control the interactions of hematopoietic cells with the vessel wall are largely undefined. Here, we report on the development of an *in vitro* 3D human marrow vascular microenvironment (VME) to study hematopoietic trafficking and the release of blood cells, specifically platelets. We show that mature megakaryocytes from aspirated marrow as well as megakaryocytes differentiated in culture from CD34+ cells can be embedded in a collagen matrix containing engineered microvessels to create a thrombopoietic VME. These megakaryocytes continue to mature, penetrate the vessel wall, and release platelets into the vessel lumen. This process can be blocked with the addition of antibodies specific for CXCR4, indicating that CXCR4 is required for megakaryocyte migration, though whether it is sufficient is unclear. The 3D marrow VME system shows considerable potential for mechanistic studies defining the role of marrow vasculature in thrombopoiesis. Through a stepwise addition or removal of individual marrow components, this model provides potential to define key pathways responsible for the release of platelets and other blood cells.

## Introduction

The adult human bone marrow releases nearly 500 billion cells into the blood each day [1,2]. Intravital imaging techniques have made it possible to visualize these complex processes in animal models, and have led to the identification of several pathways that mediate transmigration of cells from the marrow to the blood. These studies are largely conducted in rodents or zebrafish. However, the detailed interactions between the marrow vasculature and differentiated blood cells, particularly at the terminal stages of maturation and blood cell release remain elusive for human cells. There is a growing appreciation that differences in scale between man and small animals are most likely not addressed by reiterating simple three dimensional cellular relationships to compensate for increased volume. Discrepancies in kinetics and outcomes in marrow regeneration between small animals and humans underscore this point [3–6]. Therefore, *in vitro* models are needed to model these phenomena and better understand human blood cell production and release. To address this, we have developed an engineered *in vitro* platform to approximate the vascular microenvironment (VME) and examine megakaryopoiesis.

The marrow is known for its complex architecture and diverse cell types. The vasculature, adipose tissue, fibroblasts, osteoblasts, osteoclasts, and hematopoietic cells have spatial relations that are critical for ordered blood cell production [7]. Recent studies, primarily from animal models, indicate that components of the VME can play more than one role in hematopoietic regulation [8–13]. For example, the endothelium contributes signals for lineage commitment, differentiation, and mobilization of progenitors [13–15]. *In vitro* work shows that human endothelial cells specifically support the development and differentiation of myeloid and megakaryocytic progenitors [16]. In small animals, hematopoietic stem cells (HSCs) localize in the perivascular space and, as they differentiate into megakaryocytes, they are in the perfect position to release platelets into the circulation [17–21]. In large animals, the difference in scale could mean that megakaryocytes may not be restricted to perivascular spaces alone. The release of platelets into the blood vessels requires that the megakaryocytes or some part thereof to come in contact with the vessel. The model presented here suggests that megakaryocytes migrate to achieve this end.

*In vitro* studies in liquid culture have proven useful for identifying cytokines and chemokines that contribute to hematopoietic regulation, cell proliferation, maturation, and motility [22,23]. Recent studies suggest the importance of physical factors that cannot be recapitulated in liquid culture, but can be approximated in 3D cultures [24–27]. A functional VME should include at the very least, architecture defined by the fibroblast, extracellular matrix [28,29], patent vasculature, and flow to facilitate processes such as platelet shedding [24–26]. Equally important for recapitulating a marrow VME is the inclusion of relevant cell populations in appropriate spatial relationships [26,30]. Systematically addressing these components should allow for an optimized vascular platform.

**Fig 1:**
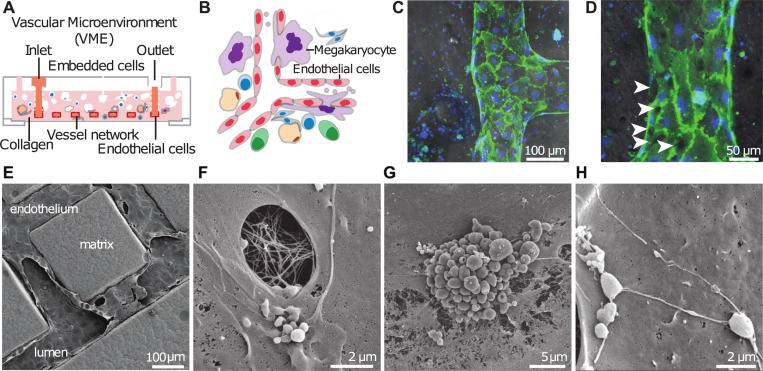
Engineered human marrow VME *in vitro*. **A.** Model schematic of marrow cells surrounding the vessel network in a collagen matrix, shown in cross section. **B.** Schematic of close-up interactions of marrow cells with vessels. **C-D.** Overlay of bright field and immunofluorescence images of a vessel surrounded by marrow cells after two weeks of culture. Green: CD31, blue: nuclei. Arrowheads in D: openings on the endothelium. **E-H.** Scanning electron microscopy shows ultrastructure of the VME. **E.** Ultrastructure of the abluminal and luminal endothelium in relation to the collagen matrix and surrounding cells. **F.** Close-up view of the vessel lumen shows pores on the endothelium. **G.** Pro-platelet-like territories and **H.** pro-platelet structures on the luminal endothelium during culture.

Here, we developed an *in vitro* microvessel system to investigate and identify critical components of the 3D marrow VME (Fig 1A and 1B). We show that megakaryocytes, either isolated from fresh human marrow aspirates or differentiated from hematopoietic progenitor cells *in vitro*, seeded into the matrix of the VME migrate over a two-week period to make contact with the vessel. Once in contact, they induce endothelial pore formation and release platelets into the lumen of the vessel. This closely approximates megakaryocyte behavior *in vivo*. Our study demonstrates the possibility of using such a 3D *in vitro* system to assemble the marrow microenvironment and examine complex hematopoietic processes.

## Experimental methods

Bone marrow aspirates were collected under FHCRC IRB protocol 0999.209 stating that leftover specimens will be stored and used for an indefinite period of time. The cells isolated from aspirates were considered non-human subjects as no identifiable information was associated with the leftover specimen. Human umbilical cord blood was purchased from Bloodworks Northwest blood bank. Fresh peripheral blood was obtained under protocols and with written consent approved by the University of Washington (Protocol: HSD 45624).

### Isolation of bone marrow aspirates and mononuclear cells

Marrow cells were obtained from discarded filters used to strain bone marrow following marrow aspiration from healthy donors in compliance with Institutional Review Board protocol, approved by the University of Washington and Fred Hutchinson Cancer Research Institute. The screen and filters containing marrow fibroblasts, stromal cells, bone pieces and fat cells were reverse perfused with 30 mL of phosphate buffered saline (PBS) and incubated for 10 min. Cells were dissociated from the screen and tubing with mechanical agitation. The resulting cell suspension was passed through a 20μm filter to separate out large cells, bone chunks, and fat globules from the smaller hematopoietic cells. Mononuclear cells, including megakaryocytes, from the filtrate were isolated through centrifugation with Ficoll-Paque (specific gravity 1.077) at 500 g for 30 minutes at room temperature. Both the unfiltered bone chunks, fat globules, large cells, and isolated bone marrow mononuclear cells (BMMCs) were resuspended and stored separately at 4C overnight in PBS with 10% fetal bovine serum (FBS) prior to use in vessel fabrication.

### Generation of megakaryocytes from human umbilical cord blood and peripheral blood

Human umbilical cord blood was purchased from BloodWorksNW blood bank. Human G-CSF mobilized peripheral blood CD34+ cells were purchased from the NIDDK-supported cell processing core at the Fred Hutchinson Cancer Research Center (CCEH; U54 DK106829). Cord blood was processed in the same method as a previous publication [31]. Hetastarch 6% wt/vol (Hospira) was added to cord blood to a final concentration of 1.2%, and cells were processed by gravity sedimentation for 60 min. The leukocyte-enriched component was separated, centrifuged for 10 min at 300g and the supernatant was removed. The cell pellet was treated with ACK lysing buffer (Invitrogen) and washed with PBS. The cells were labeled with anti-CD34 antibody conjugated to magnetic microbeads (Miltenyi Biotec). The CD34+ fraction of cells was positively selected with an autoMACS separator, yielding over 90% CD34+ cell purity, as confirmed by flow cytometry (FACSCaliber).

Human cord blood or peripheral blood CD34+ cells were differentiated to megakaryocytes as described previously [31]. CD34+ cells were plated in 6-well plates at a density of 5x104 cells/ml and cultured in serum-free X-VIVO 10 medium supplemented with a cytokine combination consisting of IL3 (10 ng/mL), IL6 (10 ng/mL), SCF (10 ng/mL) (R&D Systems), and TPO (50 ng/mL, Peprotech) [31]. The suspension cultures were incubated at 37oC in a 5% CO2 humidified chamber. Media was changed after 7 days of culture. After 10 days, cells were collected and stained with PE conjugated CD41a antibody. CD41a+ megakaryocytes were sorted at >90% purity with a BD Biosciences FACSAria III sorter.

### Washed platelets preparation

Fresh blood was drawn from healthy donors into 6mL ACD tubes (Solution B, BD Vacutainer) with written consent under protocols approved by the Institutional Review Board of the University of Washington. Washed platelets were isolated in a manner described previously [32]. Briefly, freshly drawn blood collected in 6mL ACD tubes was centrifuged at 120 x g for 15 minutes at RT medium acceleration and without brake. The platelet rich plasma (PRP) was transferred to a FACS tube using a transfer pipet. The PRP was centrifuged at 500 x g for 10 minutes at RT on slow brake and medium acceleration. The plasma was carefully removed leaving a pellet of platelets at the bottom of the tube. CGS buffer (13 mM Sodium Citrate, 120 mM Sodium Chloride,30mM Glucose, pH 6.5) was used to suspend the pellet gently and more CGS was added for a total volume of 10mL. Human recombinant PGI2 (Sigma-Aldrich) was added to the solution at 500ng/ml and the tube was inverted once to gently mix. A final centrifugation was performed at 400 x g for 10 minutes at RT with medium acceleration and slow brake. After the supernatant was removed, the platelets were resuspended in Tyrode’s Buffer at half the volume of the original PRP volume.

### Culture of human umbilical vein endothelial cells (HUVECs) and bone marrow stromal cells

HUVECs (Lonza) were cultured in endothelial cell growth media (EGM, Lonza) at 37°C in a 5% CO2 humidified chamber. HUVECs at passage 5 or 6 were used in experiments. Bone marrow stromal cell line HS5 cells were received from Dr. Beverly Torok-Storb, Fred Hutchinson Cancer Research Center, as a gift. HS5 cells were cultured in RPMI-1640 supplemented with 10% fetal bovine serum, sodium pyruvate (1 mM), L-glutamine (0.4 mg/mL), penicillin (100 U/mL), and streptomycin sulfate (100 μg/mL, Invitrogen).

### Fabrication and culture of 3D engineered microvessels and marrow VME

A microfluidic network was built via soft lithography using collagen gel (6–7.5mg/ml), as described previously [33,34]. For marrow VME studies, isolated marrow cells and globules were mixed into the collagen at approximately 10 × 10^6^ cells/mL. For permeability, migration, and maturation experiments, sorted megakaryocytes were added to the collagen at 10^6^ cells/mL, whereas for particle collection studies, unsorted megakaryocytes were added at 8–10 ×10^6^ cells/mL. Cells were thoroughly mixed into collagen yielding a uniform distribution in devices.

To seed the devices, HUVECs were trypsinized and resuspended at a concentration of 5 x 10^6^ cells/mL [33,34]. After removal of media from the inlet and outlet of the devices, 10uL of HUVEC suspension was added into the inlet of microvessel and allowed to attach at 37°C for 15 min. After attachment, media was added to the inlet reservoir for perfusion culture. In bone marrow aspirate co-cultured vessels, endothelial networks were perfused with EGM and the non-inlet/outlet reservoirs were filled with X-VIVO 10 medium supplemented with IL3 (10 ng/mL), IL6 (10 ng/mL), SCF (10 ng/mL, R&D Systems), TPO (50 ng/mL, Peprotech) and EPO (2U/mL, Affymetrix eBiosciences). In megakaryocyte vessels, endothelial networks were perfused with EGM supplemented with 100ng/ml TPO. The media for all vessels was replenished every 12 hours. In gravity driven conditions, the flow rate peaked initially at approximately 10 μL/min and decreased with time until the inlet balanced with the outlet. This range of flow rates leads to a peak wall shear stress in the inlet or outlet vessels of approximately 10 dynes/cm^2^ and an average of ~ 0.1 dynes/cm^2^ throughout the culture time.

For particle collection studies, megakaryocyte vessels were cultured under syringe pump-driven flow starting 2 days after fabrication (Model 11 Plus, Harvard Apparatus). Syringes were connected to tubing (1/32”ID, 3/32”OD Silicon Tubing, McMaster) fit securely into the inlet with a tube-to-tube 90° elbow connector. The flow rate was set at 3 μL/min so that the averaged wall shear stress in the inlet and outlet vessels remains 3 dynes/cm^2^. Perfusate was collected via outlet tubing connected to a FACS tube containing 500 μL ACD buffer (Solution B, BD Vacutainer) and 400 μL PBS (Lonza). Every 24 hours, perfusate with released particles and/or cells was collected and media was refilled.

### Functional testing and FACS

Collected particles, whole blood, and washed platelets were analyzed using flow cytometry. Half of each sample was activated with 3U/mL thrombin for 5 minutes. All samples were fixed in 3.7% formaldehyde, washed in FACS buffer (2% fetal bovine serum in PBS), and stained for CD41a, IgG (BD Biosciences), DAPI, and 7AAD (Beckman Coulter) for 30 minutes at RT. The cells were washed and analyzed on FACS CANTO2. The number of particles collected from each vessel was calculated using AccuCount Ultra Rainbow Fluorescent Particles (Spherotech). Analysis was performed on FLOWJO. Fixed quiescent and activated particles were permeabilized with Triton X-100, stained with β-tubulin (1:100, Abcam) overnight, washed, and incubated with Alexa Fluor 488 for 1 hour. Particles were washed, resuspended into a 1% agarose solution, mounted on coverslips, and imaged with a Zeiss LSM 880 confocal microscope.

### Immunofluorescence staining and confocal imaging

*In situ* fixation and immunofluorescence staining was carried out as described previously [33]. After 3 to 14 days of culture, co-cultured microvessels were fixed in situ by perfusion of 3.7% formaldehyde for 20 minutes, followed by three 15 minute washes with PBS. The devices were then perfused with blocking solution containing 2% bovine serum albumin (BSA) and 0.1% Triton X-100 (Invitrogen, Carlsbad, CA) before immunostaining. Primary antibody rabbit antihuman CD31 (Abcam), VE-Cadherin (Abcam), mouse anti-human ICAM1 (Abcam), or VCAM1 (Abcam) was diluted in the blocking solution and perfused through the vessel overnight at 4°C. The devices were washed three times with PBS for 15 minutes each. The secondary antibody goat anti-rabbit Alexa Fluor 647 or Alexa Fluor 488 (Invitrogen) and nuclear counterstain Hoechst 33342 were then perfused through the vessel for one hour, and washed three times for 20 minutes each. Immunofluorescence z-stack images (step size 1–3μm) of microvessels were taken with a Nikon A1R confocal microscope with a 10x or 20x objective. Z-projections and cross sections were generated using Image J. Zoomed views of MKs on the vessel wall and 3D reconstructions of confocal images were generated using contour surface creation in Imaris.

**Fig 2:**
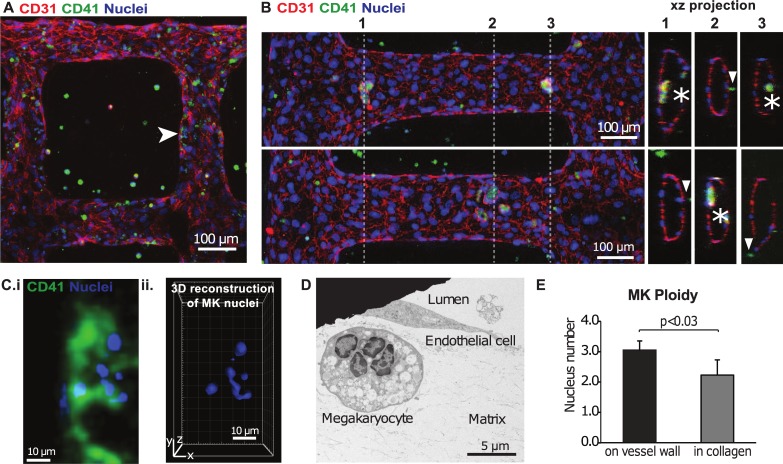
Marrow VME for the study of thrombopoiesis *in vitro*. **A**. Z-stack projection of confocal fluorescence imaging of megakaryocytes co-cultured within a 3D microvascular system. Green: CD41, red: CD31, blue: nuclei. **B**. Enlarged view, z-projection of confocal fluorescence images (left panel) and orthogonal views (right two panels) of locations at dotted lines 1 and 2, showing megakaryocytes interacting with the vessel wall (stars) and in the lumen and on the abluminal vessel wall (arrowheads). Green: CD41, red: CD31, blue: nuclei. **C**. (i) Zoomed view of megakaryocyte indicated in A (arrowhead) showing CD41a+ (green) and nucleus staining (blue) (ii) 3D reconstruction of the nucleus lobes from the megakaryocyte in i. **D**. A TEM image showing a megakaryocyte with four nucleus lobes close to a vessel. **E**. Megakaryocyte lobe counts near and far from the vessel wall shows more mature megakaryocytes are located closer to the vessel wall.

Manual quantification of megakaryocyte migration and ploidy was performed in ImageJ. Migration was quantified using 5 image stacks (120 μm depth) each from 5 different vessels, with distance from the vessel wall normalized to the radius of each vessel. The number of lobes per megakaryocyte was quantified through manual lobe counting of Hoechst-stained MK nuclei from z-projected image stacks of 6 vessels. Data are presented as mean +/- SEM. Significant differences were determined with an unpaired Student's t-test, with significance considered at p < 0.05. The number of nucleus lobes in peripheral blood-derived megakaryocytes (Fig 2) and cord blood-derived megakaryocytes (S2 Fig) were reported separately.

### Scanning electron microscopy imaging

The co-cultured devices were fixed *in situ* by perfusing 25% glutaraldehyde overnight before disassembly, where the vessel was opened to expose the luminal surface. The collagen was dehydrated in serial ethanol washes (50%, 70%, 85% and 100% ethanol) and critical point drying (Tousimis, SamDri-780). The vessels and matrices were then sputter coated with gold-palladium and analyzed by a FEI Sirion scanning electron microscope with an accelerating voltage of 5 kV, spot size 3.

### Transmission electron microscopy imaging

Microvessels were fixed in half-strength Karnovsky’s solution (2% paraformaldehyde/2.5% glutaraldehyde in 0.2 M cacodylate buffer). Microvessels were disassembled and fully immersed in the same fixative solution for several days. Samples were rinsed in 0.1 M cacodylate buffer then post-fixed using 2% OsO4 in 0.2 M cacodylate buffer followed by another rinse with 0.1 M cacodylate buffer. Sample dehydration was performed using immersions in graded solutions of ethanol, then propylene oxide (PO), before 1:1 PO/Epon 812 (Ted Pella Inc) immersion overnight. Fresh Epon 812 was then exchanged for 2 hours after which the blocks were cured for 48 hours at 60°C. Ultrathin sections (70 nm) were cut from blocks using a diamond (Diatome US) blade on a Leica EMUC6 ultra-microtome and placed onto grids. Grids were stained with uranyl acetate for 2 hours and lead citrate for 5 minutes. Sections were imaged using a JEOL JEM-1400 Transmission Electron Microscope (JEOL Ltd.) using 100 kV acceleration voltage. Images were acquired with a Gatan Ultrascan 1000XP camera (Gatan, Inc.).

### Measurement of microvessel permeability

To measure barrier function of HUVEC only, megakaryocyte co-cultured, and HUVEC with megakaryocyte-conditioned media vessels, 40kD FITC-Dextran (Sigma) was perfused through the microvessels *in situ*. Fluorescent confocal images were acquired at 1 frame/second for 10 minutes. The image sequences were analyzed with Matlab to estimate the permeability coefficient of dextran in collagen based on the model developed in a previous publication from our group [33].

## Results

### Recapitulating a 3D human marrow VME

We engineered a 3D human marrow VME in type I collagen (7.5 mg/mL) using lithographic processes described previously (Fig 1A) [33]. We embedded cells from healthy human marrow aspirates around microchannel networks in a type I collagen gel (Fig 1B). HUVECs were seeded in the lumen of microchannels within the gel and cultured under perfusion. The endothelial cells formed a single-layer vessel with cobble-stone morphology and junctions at regions of cell-cell contact (Fig 1C). Immunofluorescence images suggested that co-cultured endothelial cells were in an activated state with varying degrees of CD31 surface expression (Fig 1C and 1D) and high VCAM-1 and ICAM-1 expression (Panels D-E in S1 Fig). The vessel walls also contained openings or pores, forming a discontinuous endothelial cell layer (arrows, Fig 1D), reminiscent of pores reported on the sinusoidal marrow endothelium [35,36]. Scanning electron microscopy allowed for an ultrastructural analysis of the whole VME (Figure A in S1 Fig) and close-up views of cell-cell interactions (Fig 1E and 1H). These ultrastructural analyses confirmed the presence of pores in the endothelium, ranging from less than a micron to several microns in diameter (Fig 1F, and Panels B-C in S1 Fig). These pores only appeared in hematopoietic cell co-culture. The luminal surface contained many platelet-like structures, ostensibly derived from mature megakaryocytes seeded in the matrix, including pro-platelet clusters (Fig 1G) and string-like structures that appeared to be membrane-bound cytoplasmic beads (Fig 1H).

### Megakaryocytes migrate towards the vessel as they mature

To obtain more mechanistic insights into the differentiation of specific hematopoietic lineages, we modified the marrow VME to specifically study thrombopoiesis. Human CD34+ progenitors, isolated from either umbilical cord blood or mobilized peripheral blood, were differentiated into megakaryocytes [31]. After 10 days of culture, 30–70% of the cells expressed the megakaryocyte marker CD41a (Figure A in S2 Fig). CD41a+ megakaryocytes were then purified by flow sorting and embedded in the type I collagen gel used to cast the microvessel platform (Fig 2A) [33]. The microvessels were cultured in endothelial growth medium supplemented with thrombopoietin (TPO) under gravity or syringe pump driven flow for 3 to 14 days. Due to the network geometry, the vessel branches further away from the inlet and outlet have wall shear stress approximately fifty folds lower than the inlet and outlet [32]. The range of flow conditions (averaged to 0.1 dynes/cm^2^ in gravity driven conditions, and 3 dynes/cm^2^ under syringe pump conditions) mimics the very low wall shear stress in the small arterioles of the marrow, estimated in previous literature to range from 0 to 4.6 dynes/cm^2^ [37–39].

After 3 days of culture, endothelial cells formed junctions at regions of cell-cell contact, indicated by CD31 expression (Fig 2A and 2B). Though the majority of CD41a+ megakaryocytes remained in the matrix, some moved to the abluminal side of microvessel, and others appeared on the luminal surface of the vessel (Fig 2B). Detailed views of megakaryocytes shown interacting with the endothelial wall reveal intact, CD41a+ cells with internalized lobed nuclei (Figure E in S2 Fig). Megakaryocytes close to or in contact with the vessel wall had higher ploidy (3.1 ± 0.3SD) compared to megakaryocytes distant from the vessel (2.4 ± 0.2 SD lobes, p<0.05; Fig 2C–2E and Panels B-C in S2 Fig).

**Fig 3:**
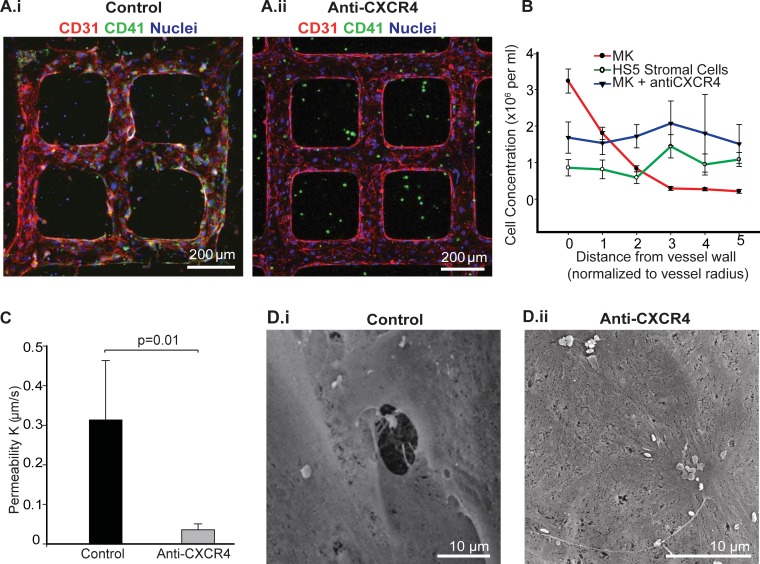
CXCR4 mediates MK migration and penetration through the endothelium. **A**. Z-stack projection of confocal fluorescence image of co-cultured thrombopoietic VME in control media (i) and media supplemented with anti-CXCR4 (ii) after three days. Green: CD41, red: CD31, and blue: nuclei. **B**. Cell density of megakaryocytes and HS5 stromal cells in collagen with respect to distance to microvessel walls after three days of culture. **C**. Permeability coefficient (mean ± S.D., n = 3) of megakaryocytes co-cultured microvessels in control and anti-CXCR4 treated conditions. **D**. SEM of microvessel lumen showing holes in the endothelium of MK vessels in control (i), but not in anti-CXCR4 supplemented conditions (ii).

After 3 days of culture, megakaryocyte density increased over three fold near the vessel wall compared to the initial seeding density of 1 million cells/mL (Fig 3A.i and 3B and Figure D in S2 Fig). Concurrently, megakaryocyte density decreased to one fifth of the original density at a distance greater than 300 μm (3 times the vessel diameter) from the vessel wall (Fig 3B), suggesting that the megakaryocytes moved towards the vessel wall. Live imaging confirmed this phenomenon, as we observed megakaryocytes in the matrix actively migrating towards the vessel wall during culture (S1 Video, S2 Video, S3 Video). Canine megakaryocytes showed the same migration pattern. Megakaryocytes isolated from dog marrow were engineered to express GFP under the PF4 promoter and cultured in the same system [40]. After 3 days of culture, the majority of these megakaryocytes closely associated with the vessel wall (Panels A-C in S3 Fig). In contrast, a control human bone marrow stromal cell line (HS5) cultured in the matrix at the same cell density showed no significant change in cell density in relation to the vessel walls throughout culture (Fig 3B).

The maturation of megakaryocytes *in vivo* is driven primarily by thrombopoietin (TPO) [19] and stromal cell-derived factor 1 (SDF-1/CXCL12) [23], among other growth factors [41]. As they mature, megakaryocytes upregulate expression of CXCR4 and respond to CXCL12 (SDF-1) signaling, which directs their migration within the hematopoietic microenvironment [20,23,24,35,42,43]. To examine the role of CXCR4/CXCL12 signaling in the migration of megakaryocytes in our system, we studied the effect of a neutralizing CXCR4 antibody. In anti-CXCR4 treated cultures, megakaryocytes remained in the matrix with no migration after three days of culture (Fig 3Aii and 3B), suggesting that CXCR4/CXCL12 signaling is necessary for megakaryocyte migration and their interactions with the microvessels.

The presence of megakaryocytes in the matrix also affected microvascular permeability. FITC-conjugated 40-kDa dextran was perfused through the microvessels to estimate the permeability coefficient *K* of the endothelium, in an approach similar to one described previously (Figure H in S3 Fig) [33]. The presence of megakaryocytes near the vessel increased microvessel permeability, with the permeability coefficient *K* = 0.31 ± 0.15 μm/s, nearly 10 fold higher than in microvessels without megakaryocytes (*K* = 0.032 ± 0.01 μm/s). Megakaryocyte conditioned media in HUVEC-only vessels decreased barrier function minimally (K = 0.11 +/- 0.03 um/s, S4 Fig). Neutralizing CXCR4 antibodies restored the barrier function of the microvessels (*K* = 0.036 ± 0.014 μm/s, p<0.05) to a value similar to that of vessels without megakaryocytes (Fig 3C) [33]. Scanning electron microscopy and confocal microscopy revealed that pores of 1–10 μm developed in the vessel wall during co-culture with megakaryocytes, similar to those seen in microvessels co-cultured with marrow aspirates. However, these pores are not seen in HUVEC-only vessels with or without conditioned media (Figure I in S3 Fig). The pores or fenestrae likely account for the increased vessel permeability (Fig 3D).

### Megakaryocytes penetrated the vessel wall and released platelet-like particles

**Fig 4:**
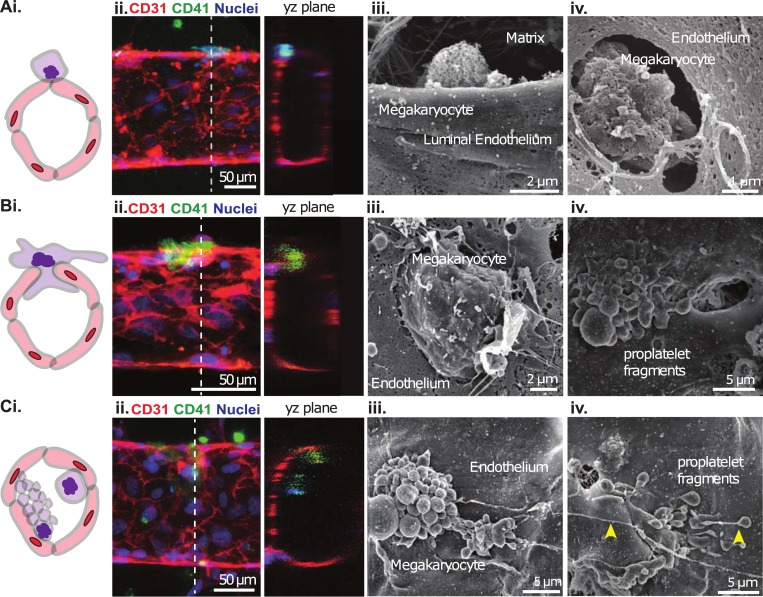
Megakaryocytes transmigrate through the endothelium and release platelet-like particles. Megakaryocytes are present in three locations relative to the microvessel wall during the process of transmigration: **A**. abluminal, **B**. transmigrating and **C**. luminal. **Column i**.: Schematics of the relative position of MKs and microvessel. **Column ii**.: z-stack projections and cross-sectional views of MK transmigration acquired through confocal fluorescence imaging. Red: CD31, green: CD41, blue: nuclei. **Columns iii-iv**.: SEM imaging of MKs interacting with the luminal endothelium at different stages, and pro-platelet fragments shedding platelet-like particles along the direction of flow (yellow arrowheads).

In the marrow, megakaryocytes must migrate across or extend processes through the vessel wall into the lumen to release platelets [18,35,44]. However, it is unclear when or how megakaryocytes transmigrate through the endothelium, mainly due to the lack of access to the marrow and complex microenvironment. Our system allowed for close-up examination of the interaction of megakaryocytes and the vessel walls in real time. Megakaryocytes were observed to develop multiple processes that extended towards the vessel wall, migrated into the lumen, and released platelet-like particles (S2 Video). Confocal and electron microscopy revealed different stages of this process (Fig 4). Some megakaryocytes resided completely on the abluminal surface of the microvessel (Fig 4A). Some other megakaryocytes occupied both the abluminal and luminal space, apparently transmigrating through the endothelium while undergoing membrane demarcation (Fig 4B) and fragmentation into clusters or pro-platelet strings on the vessel wall (Fig 4C). Large megakaryocyte fragments or whole megakaryocytes were also found on the vessel walls (S5 Fig, S6 Fig), where they would be expected to finalize their maturation and fragment into platelets while in circulation.

**Fig 5:**
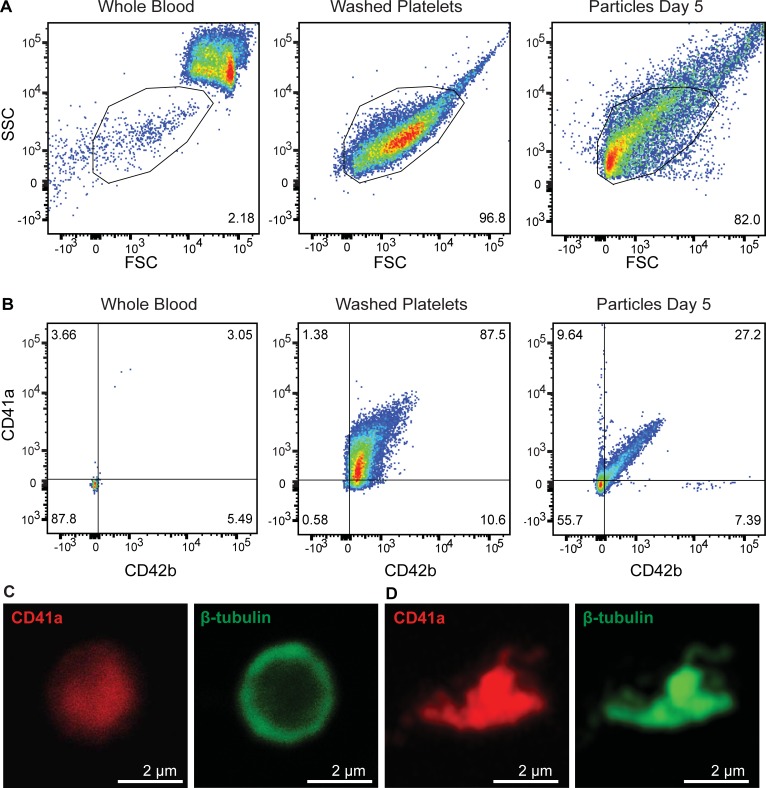
Characterization of generated platelet-like particles. **A**. Flow cytometry of whole blood, washed platelets, and collected particles from MK vessels at day five of culture showing granularity (SSC-A) and size (FSC-A). Gating was based on the platelet population of the whole blood sample. **B.** CD41a and CD42b expression of gated population from whole blood, washed platelets, and collected particles at day five. **C-D.** Immunofluorescence images of un-activated and thrombin-activated particles stained for CD41a and β-tubulin.

We next examined the capacity of our marrow VME to generate platelets. We embedded 7–10 × 10^6^/mL differentiated megakaryocytes (unsorted after 10 days of differentiation culture) in the collagen matrix for co-culture with HUVEC-lined microvessels under flow. After initial two days of culture, we collected the effluent perfusate every 24 hours (Fig 5A). The released particles/cells ranged from the size of microparticles to that of red blood cells, as defined by the forward-scatter plot of washed platelets and whole blood, along with platelet-specific CD41a and CD42b analysis (Fig 5B). The collected perfusate contained an average of 1.82 × 10^6^ platelet-sized particles, regardless of granularity, with over 550,000 CD42b+ particles in approximately 2 mL per thrombopoietic VME device per day. Each device contained microvessels with a surface area of 0.53 cm^2^ and a volume of 1.6 μL. Considering that megakaryocytes within 100 μm of the vessel wall were able to generate particles and release them into the circulation, we calculated the yield of CD42b+ platelet-sized particles per megakaryocyte per day to be approximately 27, without counting any particles adhered on the vessel wall. Cytoskeletal rearrangement was evident when particles were activated with 3U/mL thrombin for 5 minutes by immunofluorescence staining of CD41a and β-tubulin (Fig 5C and 5D).

## Discussion

The bone marrow is essentially a liquid tissue with heterogeneous population and complex architecture. Stem cell and maturing components are not ordered in recognizable spaces as they are in epithelia. This poses a problem in visualizing hematopoietic differentiation, particularly in the context of large animals. In the past decade, advances have been made to visualize thrombopoiesis in mouse models via intravital microscopy. These studies show that marrow megakaryocytes released pro-platelets into the circulation with an estimated small number of platelets per megakaryocyte to meet the need for physiological platelet turnover [18,44,45]. The process of pro-platelet formation has been recapitulated *in vitro*, and also in this case, low numbers of platelet-like particles per megakaryocyte are produced [24,26,46]. Recently, attention has been paid to finding approaches or mechanisms to enhance the number of platelets generated per megakaryocyte. Under some stress conditions, marrow megakaryocytes were found to generate very large numbers of platelets to meet acute platelet need, potentially releasing them through rupture within the blood vessels, though this phenomena is under debate [21,47].

Here, we used hydrogel matrices composed of 0.75% type I collagen and 99.25% water to reconstruct part of marrow tissue and study the close interactions between megakaryocytes and microvessel walls. We showed that megakaryocytes from freshly aspirated marrow modified the endothelium of microvessels, interacted with vessel walls, and released platelet-like particles into the lumen. Although this system can benefit from further optimization and biological analysis, it validates the approach of using an engineered microvessel system to model the human marrow VME. We embedded purified CD41+ megakaryocytes in the engineered marrow VME, and for the first time, detailed the specific interactions of megakaryocytes with the vessel wall. The combination of three types of imaging data, namely live imaging, immunofluorescence, and scanning electron microscopic images, confirmed that megakaryocytes migrated towards the vessel and created pores in the endothelium, through which they either transmigrated or extended pro-platelets into the vessel lumen to release platelets. We showed that HUVECs, known to form continuous endothelial surfaces in both large vessels [48] and within *in vitro* microvessels [33], were modified by the surrounding megakaryocytes to become discontinuous and leaky. This structural phenotype is found in bone marrow endothelial cells, which are reported to be highly fenestrated [23,43,49–52]. The mechanisms behind MK-induced pore formation have been suggested to arise from the matrix-degrading properties of the MK podosome, which are increased in the presence of endothelial basement membrane and SDF1 [53–55] In contrast, megakaryocyte conditioned media alone did not change the continuity of HUVEC microvessels. This suggests that the microenvironment can modulate specific endothelial cell phenotypes to adapt to local functional needs, which may be one source of endothelial cell heterogeneity [56,57].

We showed that megakaryocyte migration is mediated in part by CXCR4-CXCL12 signaling, as blocking CXCR4 led to reduced megakaryocyte migration. Though not the only chemoattractant responsible for MK migration in vivo, this platform provides opportunities to further investigate and modulate the paracrine signaling and cell-cell interactions in thrombopoiesis, particularly in a system that allows for the migration of cells from large distances away from vasculature. In addition, our system allows for the precise control of flow and perfusion of biochemical cytokines through the microvessels and particle collection from the outlets. Throughout our culture, flow has been maintained by gravity or syringe pump. The flow rate and resulting wall shear stress mimics a biologically relevant range, which has been reported as extremely low, yielding wall shear stress range between 0–4.6 dynes/cm^2^ [37–39].

We showed different processes of platelet release, namely pro-platelet territories, platelet-like blebs, and large fragments found in the vessel lumen. Mature megakaryocytes normally release pro-platelets, which are long cytoplasmic extensions with a beaded structure that fragment into platelets in circulation [18,44,58]. We see CD41+ megakaryocytes localize around the vessel wall and extend processes through the endothelium to release particles. The generated platelet-like particles found on the vessel lumen or collected have similar morphology as seen via ultrastructure assessment from electron microscopy and marker expression from immunofluorescence microscopy. Though Nishimura et al show that megakaryocytes can rupture in the presence of inflammatory cues to meet acute platelet need, this phenomena is not widely observed [47,59]. Recent work by Itkin et al. identified different roles of distinct marrow blood vessel types in hematopoiesis: less permeable arterial vessels support a low reactive oxygen species environment, whereas more permeable marrow sinusoids promote the activation of hematopoietic progenitor cells and immature cell trafficking [15]. In our system, endothelial cells appear to be activated and display structural and functional changes, indicating an inflammatory environment. Megakaryocyte-induced permeability could provide sites for immature megakaryocytes or large fragments to migrate across the endothelium, in combination with the lack of mature basement membrane formation. In normal marrow, whole megakaryocytes typically do not transmigrate from the marrow into the venous blood *in vivo*. This may impact the release of megakaryocytes and/or platelets, leading to the multiple types of megakaryocyte fragmentation phenomena observed in our system.

The advantages of our system include controllable cellular composition, matrix, vascular structure, flow, and a 3D geometry, and preserves the capacity for high quality imaging. Nevertheless, there are still improvements that can address technical limitations present in our platform. For example, the vessel wall consists of a single layer of endothelium with a 100 μm diameter, which is larger than the marrow sinusoids. This diameter may induce different stresses and biophysical forces on the vessel walls and affect the cellular interactions between megakaryocytes and the endothelium. Future studies could examine the effect of vessel diameter on thrombopoiesis, ranging from 30 μm, the lower limit of our fabrication technique, to sub millimeter or examine the impact of vessel density on megakaryocyte migration. In addition, we observed large numbers of platelet-like particles adhered to the luminal wall, rather than flowing in the perfused media. It is unclear whether this occurs *in vivo*, and if so, whether physiological mechanisms exist to detach the platelets. It is possible that such adhesion may be an *in vitro* artifact due to high serum content in the media, which activates platelets after their release. In addition, the endothelial cells appeared to have become activated and fenestrated during culture. It would be interesting to re-examine this phenomenon with bone marrow sinusoid endothelial cells or endothelial cells that do not require serum *in vitro* to better mimic the marrow vasculature [60]. It is also expected that bone marrow endothelial cells have a unique phenotype, representing a less inflammatory and adhesive surface [15,61]. Adhesion blocking reagents can also be introduced through the vessel wall to allow for better release of particles. Finally, our collected platelet-like particles appeared to lack packaged granules present in human platelets, which could be improved by addition of blood proteins in the media or additional matrix components in biologically relevant gradients, such as laminin, fibronectin, or collagen IV. The production of proper granules could also lead to a more typical morphology in activated particles. These modifications to the culture system could improve the shortcomings of a collagen-based platform and yield more homogenous particles without pre-activation. Nevertheless, our system demonstrated a functional *in vitro* human marrow VME that lends itself to future mechanistic studies on cell-cell and cell-matrix interactions in the marrow.

## Supporting information

S1 Fig Engineered human marrow VME in vitro. Scanning electron microscopy shows ultrastructure of the VME. A. A stitched large image acquisition showing the entire vessel network in the VME after two weeks of culture. B. SEM image of the endothelium of the bone marrow cell co-cultured vessels showing holes from abluminal side. C. SEM image of the luminal side of the endothelium with no co-cultured cells shows an intact, continuous endothelium. D-E. Immunofluorescence staining for (D) ICAM-1 and (E.i.) VCAM-1 in the human marrow VME and in a (ii) HUVEC-only culture.
(EPS)

S2 Fig Megakaryocytes differentiation and interaction with microvessels. A. An example flow analysis showing CD34+ cells after day 9 of differentiation in suspension had approximately 36% CD41+ megakaryocytes. Unstained population in gray, CD41+ stained cells in green. B. (i) TEM image of MKs far from the vessel wall within the collagen matrix shows low nucleus lobe number. (ii) 3D reconstruction of confocal image from a megakaryocyte nucleus 3 radius-lengths from the vessel wall also shows low nucleus lobe number. C. Quantification of nucleus lobe number for peripheral blood-derived and cord blood-derived MKs on the vessel wall and far from the vessel wall shows decreased lobe number in cord-blood derived MKs. D.i-iii. Human MKs line the vessel walls after 3 days of culture. E. (i) Zoomed view of MKs shown in cross section in Fig 2B confirm the CD41a+ cells contain nuclei. Cells shown are from (i) cross section 1 and (ii) 3 from the top panel, and cross section (iii) 2 and (iv) 3 from the bottom panel.
(EPS)

S3 Fig Canine PF4-GFP megakaryocytes migrate to the vessel wall. A. MKs from canine marrow with PF4-driven GFP expression were isolated and seeded into the collagen matrix surrounding the microvessels. Canine MKs, similar to human MKs, migrated to the vessel wall after 3 days of culture. B. Quantification of canine MK distance from the vessel shows a nearly five-fold increase in MK concentration at the wall. Error bars indicate standard error. C. SEM images of the endothelium in co-culture with canine MKs shows a pore in the vessel wall with an MK behind it (i) and a pro-platelet cluster with MK fragments on the endothelium (ii).
(EPS)

S4 Fig Endothelial barrier function in co-cultured vessels. A. 40kD-FITC-Dextran was perfused through the MK co-cultured (control) vessels (i) and MK vessels treated with antiCXCR4 (ii) to visualize barrier function of the vessels. (iii) FITC-Dextran was als perfused through vessels fed with MK-conditioned media to test barrier function. B. Junctional staining (i) and scanning electron microscopy (ii) do not show holes in MK-conditioned media cultured vessels.
(EPS)

S5 Fig Whole megakaryocytes penetrate into the vessel lumen. A-C. Scanning electron microscopy of a human thrombopoietic VME shows ultrastructure of whole megakaryocytes or large fragments within the vessel lumen.
(EPS)

S6 Fig Flow Cytometry Controls for generated particles. A. Corresponding plots to whole blood, washed platelet, and generated particle CD41a and CD42b staining show unstained populations of plots in Fig 5.
(EPS)

S1 Video Live imaging of megakaryocytes in the matrix migrating towards the vessel wall during culture. (MP4)

S2 Video Live imaging of megakaryocytes in the matrix developing multiple processes that extended towards the vessel wall, migrated into the lumen, and released platelet-like particles. (MP4)

S3 Video Live imaging of megakaryocytes in the matrix migrating towards the vessel wall, migrated into the lumen. (MP4)

## References

[1] Fliedner TMM, Graessle D, Paulsen C, Reimers K. Structure and function of bone marrow hemopoiesis: mechanisms of response to ionizing radiation exposure. Cancer Biother Radiopharm. 2002;17: 405–426. doi: 10.1089/108497802760363204 12396705 10.1089/108497802760363204

[2] Orkin SH, Zon LI. Hematopoiesis: An Evolving Paradigm for Stem Cell Biology. Cell. 2008;132: 631–644. doi: 10.1016/j.cell.2008.01.025 18295580 10.1016/j.cell.2008.01.025 PMC2628169

[3] Dominietto A, Raiola AM, Van Lint MT, Lamparelli T, Gualandi F, Berisso G, et al Factors influencing haematological recovery after allogeneic haemopoietic stem cell transplants: graft‐versus‐host disease, donor type, cytomegalovirus infections and cell dose. Br J Haematol. Blackwell Science Ltd; 2001;112: 219–227. doi: 10.1046/J.1365-2141.2001.02468.X 11167808 10.1046/j.1365-2141.2001.02468.x

[4] Ramírez P, Brunstein CG, Miller B, DeFor T, Weisdorf D. Delayed platelet recovery after allogeneic transplantation: a predictor of increased treatment-related mortality and poorer survival. Bone Marrow Transplant. Nature Publishing Group; 2011;46: 981–986. doi: 10.1038/bmt.2010.218 20921943 10.1038/bmt.2010.218

[5] Zijlmans JM, Visser JW, Laterveer L, Kleiverda K, Heemskerk DP, Kluin PM, et al The early phase of engraftment after murine blood cell transplantation is mediated by hematopoietic stem cells. Proc Natl Acad Sci U S A. National Academy of Sciences; 1998;95: 725–9. Available: http://www.ncbi.nlm.nih.gov/pubmed/9435260 9435260 10.1073/pnas.95.2.725 PMC18488

[6] Uchida N, Tsukamoto A, He D, Friera AM, Scollay R, Weissman IL. High doses of purified stem cells cause early hematopoietic recovery in syngeneic and allogeneic hosts. J Clin Invest. 1998;101: 961–966. doi: 10.1172/JCI1681 9486965 10.1172/JCI1681 PMC508646

[7] Morrison SJ, Scadden DT. The bone marrow niche for haematopoietic stem cells. Nature. 2014;505: 327–34. doi: 10.1038/nature12984 24429631 10.1038/nature12984 PMC4514480

[8] Arai F, Hirao A, Ohmura M, Sato H, Matsuoka S, Takubo K, et al Tie2/angiopoietin-1 signaling regulates hematopoietic stem cell quiescence in the bone marrow niche. Cell. 2004;118: 149–161. doi: 10.1016/j.cell.2004.07.004 15260986 10.1016/j.cell.2004.07.004

[9] Butler JM, Nolan DJ, Vertes EL, Varnum-Finney B, Kobayashi H, Hooper AT, et al Endothelial cells are essential for the self-renewal and repopulation of Notch-dependent hematopoietic stem cells. Cell Stem Cell. 2010;6: 251–64. doi: 10.1016/j.stem.2010.02.001 20207228 10.1016/j.stem.2010.02.001 PMC2866527

[10] Ding L, Saunders TL, Enikolopov G, Morrison SJ. Endothelial and perivascular cells maintain haematopoietic stem cells. Nature. Nature Publishing Group; 2012;481: 457–462. doi: 10.1038/nature10783 22281595 10.1038/nature10783 PMC3270376

[11] Doan PL, Russell JL, Himburg HA, Helms K, Harris JR, Lucas J, et al Tie2(+) bone marrow endothelial cells regulate hematopoietic stem cell regeneration following radiation injury. Stem Cells. 2013;31: 327–37. doi: 10.1002/stem.1275 23132593 10.1002/stem.1275 PMC3580267

[12] Kunisaki Y, Bruns I, Scheiermann C, Ahmed J, Pinho S, Zhang D, et al Arteriolar niches maintain haematopoietic stem cell quiescence. Nature. Nature Publishing Group; 2013;502: 637–643. doi: 10.1038/nature12612 24107994 10.1038/nature12612 PMC3821873

[13] Wang LD, Wagers AJ. Dynamic niches in the origination and differentiation of haematopoietic stem cells. Nat Rev Mol Cell Biol. Nature Publishing Group, a division of Macmillan Publishers Limited. All Rights Reserved.; 2011;12: 643–55. doi: 10.1038/nrm3184 21886187 10.1038/nrm3184 PMC4040463

[14] Gori JL, Butler JM, Chan Y-Y, Chandrasekaran D, Poulos MG, Ginsberg M, et al Vascular niche promotes hematopoietic multipotent progenitor formation from pluripotent stem cells. J Clin Invest. 2015;125: 1243–54. doi: 10.1172/JCI79328 25664855 10.1172/JCI79328 PMC4362238

[15] Itkin T, Gur-Cohen S, Spencer JA, Schajnovitz A, Ramasamy SK, Kusumbe AP, et al Distinct bone marrow blood vessels differentially regulate haematopoiesis. Nature. Nature Publishing Group, a division of Macmillan Publishers Limited. All Rights Reserved.; 2016;advance on. doi: 10.1038/nature17624 10.1038/nature17624 PMC6450701 27074509

[16] Rafii S, Shapiro F, Pettengell R, Ferris B, Nachman RL, Moore M a, et al Human bone marrow microvascular endothelial cells support long-term proliferation and differentiation of myeloid and megakaryocytic progenitors. Blood. 1995;86: 3353–63. Available: http://www.bloodjournal.org/content/86/9/3353.abstract 7579438

[17] Huang H, Cantor AB. Common features of megakaryocytes and hematopoietic stem cells: what’s the connection? J Cell Biochem. 2009;107: 857–64. doi: 10.1002/jcb.22184 19492306 10.1002/jcb.22184 PMC2741141

[18] Junt T, Schulze H, Chen Z, Massberg S, Goerge T, Krueger A, et al Dynamic visualization of thrombopoiesis within bone marrow. Science. 2007;317: 1767–1770. doi: 10.1126/science.1146304 17885137 10.1126/science.1146304

[19] Kaushansky K, Broudy VC, Lin N, Jorgensen MJ, McCarty J, Fox N, et al Thrombopoietin, the Mp1 ligand, is essential for full megakaryocyte development. Proc Natl Acad Sci. 1995;92: 3234–3238. doi: 10.1073/pnas.92.8.3234 7536928 10.1073/pnas.92.8.3234 PMC42140

[20] Machlus KR, Italiano JE. The incredible journey: From megakaryocyte development to platelet formation. J Cell Biol. 2013;201: 785–796. doi: 10.1083/jcb.201304054 23751492 10.1083/jcb.201304054 PMC3678154

[21] Stegner D, VanEeuwijk JMM, Angay O, Gorelashvili MG, Semeniak D, Pinnecker J, et al Thrombopoiesis is spatially regulated by the bone marrow vasculature. Nat Commun. 2017;8: 127 doi: 10.1038/s41467-017-00201-7 28743899 10.1038/s41467-017-00201-7 PMC5527048

[22] Gartner S, Kaplan HS. Long-term culture of human bone marrow cells. Proc Natl Acad Sci U S A. 1980;77: 4756–9. Available: http://www.pubmedcentral.nih.gov/articlerender.fcgi?artid=349925&tool=pmcentrez&rendertype=abstract 6933522 10.1073/pnas.77.8.4756 PMC349925

[23] Hamada T, Möhle R, Hesselgesser J, Hoxie J, Nachman RL, Moore MAS, et al Transendothelial migration of megakaryocytes in response to stromal cell-derived factor 1 (SDF-1) enhances platelet formation. J Exp Med. 1998;188: 539–48. doi: 10.1084/jem.188.3.539 9687531 10.1084/jem.188.3.539 PMC2212480

[24] Di Buduo CA, Wray LS, Tozzi L, Malara A, Chen Y, Ghezzi CE, et al Programmable 3D silk bone marrow niche for platelet generation ex vivo and modeling of megakaryopoiesis pathologies. Blood. American Society of Hematology; 2015;125: 2254–2264. doi: 10.1182/blood-2014-08-595561 25575540 10.1182/blood-2014-08-595561 PMC4383799

[25] Thon JN, Dykstra BJ, Beaulieu LM. Platelet bioreactor: accelerated evolution of design and manufacture. Platelets. Taylor & Francis; 2017;28: 472–477. doi: 10.1080/09537104.2016.1265922 28112988 10.1080/09537104.2016.1265922 PMC5507711

[26] Thon JN, Mazutis L, Wu S, Sylman JL, Ehrlicher A, Machlus KR, et al Platelet bioreactor-on-a-chip. Blood. American Society of Hematology; 2014;124: 1857–1867. doi: 10.1182/blood-2014-05-574913 25606631 10.1182/blood-2014-05-574913 PMC4168343

[27] Di Maggio N, Piccinini E, Jaworski M, Trumpp A, Wendt DJ, Martin I. Toward modeling the bone marrow niche using scaffold-based 3D culture systems. Biomaterials. Elsevier Ltd; 2011;32: 321–329. doi: 10.1016/j.biomaterials.2010.09.041 20952054 10.1016/j.biomaterials.2010.09.041

[28] Choi JS, Mahadik BP, Harley BAC. Engineering the hematopoietic stem cell niche: Frontiers in biomaterial science. Biotechnol J. 2015;10: 1529–1545. doi: 10.1002/biot.201400758 26356030 10.1002/biot.201400758 PMC4724421

[29] Torisawa Y, Spina CS, Mammoto T, Mammoto A, Weaver JC, Tat T, et al Bone marrow-on-a-chip replicates hematopoietic niche physiology in vitro. Nat Methods. 2014;11: 663–9. doi: 10.1038/nmeth.2938 24793454 10.1038/nmeth.2938

[30] Malara A, Gruppi C, Pallotta I, Spedden E, Tenni R, Raspanti M, et al Extracellular matrix structure and nano-mechanics determine megakaryocyte function. Blood. 2011;118 Available: http://www.bloodjournal.org/content/118/16/4449.long?sso-checked=true 10.1182/blood-2011-04-345876 PMC3291488 21828129

[31] Sun S, Wang W, Latchman Y, Gao D, Aronow B, Reems J-A. Expression of plasma membrane receptor genes during megakaryocyte development. Physiol Genomics. 2013;45: 217–227. doi: 10.1152/physiolgenomics.00056.2012 23321270 10.1152/physiolgenomics.00056.2012 PMC3615580

[32] Zheng Y, Chen J, López JA. Flow-driven assembly of VWF fibres and webs in in vitro microvessels. Nat Commun. 2015;6: 7858 doi: 10.1038/ncomms8858 26223854 10.1038/ncomms8858 PMC4522708

[33] Zheng Y, Chen J, Craven M, Choi NW, Totorica S, Diaz-Santana A, et al In vitro microvessels for the study of angiogenesis and thrombosis. Proc Natl Acad Sci U S A. 2012;109: 9342–7. doi: 10.1073/pnas.1201240109 22645376 10.1073/pnas.1201240109 PMC3386137

[34] Roberts MA, Kotha SS, Phong KT, Zheng Y. Micropatterning and Assembly of 3D Microvessels. J Vis Exp. 2016; e54457–e54457. doi: 10.3791/54457 27685466 10.3791/54457 PMC5092002

[35] Avecilla ST, Hattori K, Heissig B, Tejada R, Liao F, Shido K, et al Chemokine-mediated interaction of hematopoietic progenitors with the bone marrow vascular niche is required for thrombopoiesis. Nat Med. 2004;10: 64–71. doi: 10.1038/nm973 14702636 10.1038/nm973

[36] Becker RP, De Bruyn PP. The transmural passage of blood cells into myeloid sinusoids and the entry of platelets into the sinusoidal circulation; a scanning electron microscopic investigation. Am J Anat. 1976;145: 183–205. doi: 10.1002/aja.1001450204 1258805 10.1002/aja.1001450204

[37] nemark P-I. Vital microscopy of bone marrow in rabbit. Scand J Clin Lab Invest. 1959;38: 1–82. Available: http://www.worldcat.org/title/vital-microscopy-of-bone-marrow-in-rabbit/oclc/14623239 13658913

[38] Brookes M, Revell WJ. Blood Supply of Bone: Scientific Aspects. Springer London; 1998.

[39] Mazo IB, Gutierrez-Ramos JC, Frenette PS, Hynes RO, Wagner DD, von Andrian UH. Hematopoietic progenitor cell rolling in bone marrow microvessels: parallel contributions by endothelial selectins and vascular cell adhesion molecule 1. J Exp Med. 1998;188: 465–74. doi: 10.1084/jem.188.3.465 9687524 10.1084/jem.188.3.465 PMC2212463

[40] Kufrin D, Eslin DE, Bdeir K, Murciano J-C, Kuo A, Kowalska MA, et al Antithrombotic thrombocytes: ectopic expression of urokinase-type plasminogen activator in platelets. Blood. 2003;102 Available: http://www.bloodjournal.org/content/102/3/926.long?sso-checked=true 10.1182/blood-2003-01-0054 12689937

[41] Kuter DJ. New thrombopoietic growth factors [Internet]. Blood. 2007 pp. 4607–4616. doi: 10.1182/blood-2006-10-019315 17289815 10.1182/blood-2006-10-019315 PMC1885525

[42] Pang L, Weiss MJ, Poncz M. Megakaryocyte biology and related disorders. J Clin Invest. American Society for Clinical Investigation; 2005;115: 3332–8. doi: 10.1172/JCI26720 16322777 10.1172/JCI26720 PMC1297258

[43] Pallotta I, Lovett M, Kaplan DL, Balduini A. Three-Dimensional System for the *In Vitro* Study of Megakaryocytes and Functional Platelet Production Using Silk-Based Vascular Tubes. Tissue Eng Part C Methods. Mary Ann Liebert, Inc. 140 Huguenot Street, 3rd Floor New Rochelle, NY 10801 USA; 2011;17: 1223–1232. doi: 10.1089/ten.tec.2011.0134 21895494 10.1089/ten.tec.2011.0134 PMC3226422

[44] Zhang L, Urtz N, Gaertner F, Legate KR, Petzold T, Lorenz M, et al Sphingosine kinase 2 (Sphk2) regulates platelet biogenesis by providing intracellular sphingosine 1-phosphate (S1P). Blood. 2013;122: 791–802. doi: 10.1182/blood-2012-12-473884 23775711 10.1182/blood-2012-12-473884

[45] Vinet L, Zhedanov A. A “missing” family of classical orthogonal polynomials. J Exp Med. 2010;209: 2165–81. doi: 10.1088/1751-8113/44/8/085201

[46] Thon JN, Macleod H, Begonja AJ, Zhu J, Lee K-C, Mogilner A, et al Microtubule and cortical forces determine platelet size during vascular platelet production. Nat Commun. Nature Publishing Group, a division of Macmillan Publishers Limited. All Rights Reserved.; 2012;3: 852 doi: 10.1038/ncomms1838 22617292 10.1038/ncomms1838

[47] Nishimura S, Nagasaki M, Kunishima S, Sawaguchi A, Sakata A, Sakaguchi H, et al IL-1α induces thrombopoiesis through megakaryocyte rupture in response to acute platelet needs. J Cell Biol. 2015;209: 453–466. doi: 10.1083/jcb.201410052 25963822 10.1083/jcb.201410052 PMC4427781

[48] Baudin B, Bruneel A, Bosselut N, Vaubourdolle M. A protocol for isolation and culture of human umbilical vein endothelial cells. Nat Protoc. Nature Publishing Group; 2007;2: 481–485. doi: 10.1038/nprot.2007.54 17406610 10.1038/nprot.2007.54

[49] Kopp H-G, Avecilla ST, Hooper AT, Rafii S. The bone marrow vascular niche: home of HSC differentiation and mobilization. Physiology (Bethesda). 2005;20: 349–356. doi: 10.1152/physiol.00025.2005 16174874 10.1152/physiol.00025.2005

[50] Malara A, Currao M, Gruppi C, Celesti G, Viarengo G, Buracchi C, et al Megakaryocytes Contribute to the Bone Marrow-Matrix Environment by Expressing Fibronectin, Type IV Collagen, and Laminin. Stem Cells. 2014;32: 926–937. doi: 10.1002/stem.1626 24357118 10.1002/stem.1626 PMC4096110

[51] Hooper AT, Butler JM, Nolan DJ, Kranz A, Iida K, Kobayashi M, et al Engraftment and Reconstitution of Hematopoiesis Is Dependent on VEGFR2-Mediated Regeneration of Sinusoidal Endothelial Cells. Cell Stem Cell. Elsevier; 2009;4: 263–274. doi: 10.1016/j.stem.2009.01.006 19265665 10.1016/j.stem.2009.01.006 PMC3228275

[52] Hagiwara T, Nagasawa T, Nagahisa H, Takizawa M, Osada M, Abe T. Expression of adhesion molecules on cytoplasmic processes of human megakaryocytes. Exp Hematol. 1996;24: 690–5. Available: http://www.ncbi.nlm.nih.gov/pubmed/8635524 8635524

[53] Sabri S, Foudi A, Boukour S, Franc B, Charrier S, Jandrot-Perrus M, et al Deficiency in the Wiskott-Aldrich protein induces premature proplatelet formation and platelet production in the bone marrow compartment. Blood. American Society of Hematology; 2006;108: 134–40. doi: 10.1182/blood-2005-03-1219 16522820 10.1182/blood-2005-03-1219

[54] French DL. Megakaryocytes put a foot through the door. Blood. 2013;121: 2379–80. doi: 10.1182/blood-2013-02-479022 23538231 10.1182/blood-2013-02-479022

[55] Schachtner H, Calaminus SDJ, Sinclair A, Monypenny J, Blundell MP, Leon C, et al Megakaryocytes assemble podosomes that degrade matrix and protrude through basement membrane. Blood. American Society of Hematology; 2013;121: 2542–52. doi: 10.1182/blood-2012-07-443457 23305739 10.1182/blood-2012-07-443457

[56] Aird WC. Phenotypic heterogeneity of the endothelium: I. Structure, function, and mechanisms. Circ Res. 2007;100: 158–73. doi: 10.1161/01.RES.0000255691.76142.4a 17272818 10.1161/01.RES.0000255691.76142.4a

[57] Nolan D, Ginsberg M, Israely E, Palikuqi B, Poulos MG, James D, et al Molecular Signatures of Tissue-Specific Microvascular Endothelial Cell Heterogeneity in Organ Maintenance and Regeneration. Dev Cell. Elsevier Inc.; 2013;26: 204–219. doi: 10.1016/j.devcel.2013.06.017 23871589 10.1016/j.devcel.2013.06.017 PMC3873200

[58] Italiano JE, Patel-Hett S, Hartwig JH. Mechanics of proplatelet elaboration. J Thromb Haemost. 2007;5 Suppl 1: 18–23. doi: 10.1111/j.1538-7836.2007.02487.x 17635704 10.1111/j.1538-7836.2007.02487.x

[59] Dütting S, Gaits-Iacovoni F, Stegner D, Popp M, Antkowiak A, van Eeuwijk JMM, et al A Cdc42/RhoA regulatory circuit downstream of glycoprotein Ib guides transendothelial platelet biogenesis. Nat Commun. 2017;8: 15838 doi: 10.1038/ncomms15838 28643773 10.1038/ncomms15838 PMC5481742

[60] Seandel M, Butler JM, Kobayashi H, Hooper AT, White IA, Zhang F, et al Generation of a functional and durable vascular niche by the adenoviral E4ORF1 gene. Proc Natl Acad Sci U S A. 2008;105: 19288–93. doi: 10.1073/pnas.0805980105 19036927 10.1073/pnas.0805980105 PMC2588414

[61] Schweitzer CM, van der Schoot CE, Dräger AM, van der Valk P, Zevenbergen A, Hooibrink B, et al Isolation and culture of human bone marrow endothelial cells. Exp Hematol. 1995;23: 41–8. Available: http://www.ncbi.nlm.nih.gov/pubmed/7995370 7995370

